# The Art of Longevity

**Published:** 1897-08-28

**Authors:** 


### The Hospital

A Journal of

### The Medical Sciences and Hospital Administration.

Vol. XXII.—No. 570.] [Aug. 28, 1897.

### The Art of Longevity.

The recent death of Sir Isaac Holden, at the advanced age of ninety-one years, has induced a daily contemporary to devote a column of its space to an article on the Art of Longevity of a kind which seems to imply that the long life of the deceased gentleman might be expected to fall to the lot of any others who would pursue a somewhat similar regimen. We are reminded of a case tried before the late Mr. Justice Denman about a disputed right of way in an agricultural parish, which mainly turned upon the recollections of the oldest inhabitants. One of these was a hale and vigorous yeoman of eighty-five, whose erect figure, keen intelligence, strong voice, and clear testimony created so strong a sensation in the court that the Judge, who was by no means free from a tendency to improve any promising occasion, detained him in the box after his evidence on the matters in dispute had been concluded, and questioned him concerning his mode of life. The witness, nothing loth, explained that he was a vegetarian and a total abstainer, and entered into various details concerning his hours of rising and of retiring, the amount and character of his daily exercise, and other matters of like kind. The Judge, in dismissing him, expressed a hope that all who were present might profit by his example, and then the next witness was called. This was another yeoman, the elder brother of the preceding, and fully a match for him in strength, activity, and intelligence. As he was about to retire the Judge stopped him, with the observation, "I presume that you also, Mr. Greenfield, are indebted for the preservation of your strength and faculties to a careful observance of the same sobriety and of the same regimen which have been so well described to us by your brother?" "Ha'n't been to bed sober for fifty years, my lord," was the unblushing and startlingly unexpected reply.

It seems to be true, as far as the histories of recorded cases enable us to judge, that great longevity has been attained under such various conditions of living as to render it impossible to attach much importance to any of them. If there be any single character common to long livers in general, or to the majority of them, it is probably the avoidance of excess—or rather the habitual practice of moderation—in eating. The old saying, that men dig their graves with their teeth, has manifestly a basis of truth underlying it, and it is possible that the art of the dentist, extolled though it has been as a factor contributory to length of days, may sometimes, especially in the moderately aged, exert an opposite influence by allowing the digging to be done more effectively. However this may be, it is at least certain that Sir Isaac Holden was a very moderate eater, and Cornaro, who also lived to a great age, carried frugality of diet to the brink of starvation. But it must probably be assumed that length of days in most cases is largely dependent upon some inherent peculiarity in the organism, by which it is enabled to exist for a period beyond the average. Long life is often hereditary in families, and may be a characteristic of them for many generations; but, apart from this, we should be inclined to say that one of its most constant factors is habitual tranquility of mind, a state which may be arrived at by very different paths. There is what may be called the vegetative tranquility of the unlettered poor, of which Austin sings:—

> "The canker comes on the garden rose, and not on the wilding briar.
> Doubt and gloom are not theirs, and so they but work and love; they live,
> Rich in the only valid boons that life can withhold or give."

Everyone with experience of the poor, more especially in provincial towns and country districts, can cite examples of this class, and in them the prolongation of life is probably due in great measure, if not entirely, to the economy with which its operations are conducted. The lamp gives little light, but as a compensation it consumes only a little oil. There is, on the other hand, the tranquility which springs from the habitual exercise of the higher mental faculties, the faculties of observation, of comparison, and of judgment. South, in his sermon on the character of Adam, tells us that the common ancestor of the human race would not be concerned about things which he saw through; and the man of philosophical mind, he who habitually refers effects to their causes, may be said to see through a large proportion of the events which are antagonistic to the tranquility of the majority. Great mathematicians have generally been long lived; and the exceptions have been due to causes, such as tuberculosis, which in no way affect the validity of the rule. Great poets have been long lived—of course, with exceptions of a similar kind. Byron and Shelley were practically the victims of accident; Scott, to those who read between the lines of Lockhart's Life by the light of modern science, was manifestly sacrificed to the combined influences of excessive work and of excessive blood-letting. Great lawyers have been long lived, in spite of days spent in the fetid and exhausted atmosphere of law courts, of nights often divided between study and conviviality, and sometimes, at least, of habits which would not commend themselves either to the rigid moralist or to the enthusiastic total abstainer. Lord Eldon, for example, is said to have drank a bottle of port every weekday for many years; and every Sunday his brother, Lord Stowell, dined with him, and they each drank two. They each lived to be a good deal past eighty. The lives of great lawyers are prolonged by the necessity of constantly mastering new details in the actions which come before them. The higher faculties of their minds are kept active and recipient, and their brains keep them alive when their physical powers begin to decay. In many businesses, on the other hand, the man learns some definite procedure by the time he is thirty, and afterwards does nothing more than plod along the grooves of an habitual routine. When his powers decay, there is nothing left to live. His death will be attributed to the particular change by which it is immediately preceded, to heart, to gout, or to liver; but it will be indirectly attributable in many instances to absence of mental cultivation and of intellectual effort. Our advice to those who desire longevity would be to eat sparingly; and, unless their business—like that of a lawyer—demands constant exercise of the higher mental faculties, to study some abstruse question in such intervals of leisure as they can obtain.

